# Brazil's health system functionality amidst of the COVID-19 pandemic: An analysis of resilience

**DOI:** 10.1016/j.lana.2022.100222

**Published:** 2022-03-05

**Authors:** Alessandro Bigoni, Ana Maria Malik, Renato Tasca, Mariana Baleeiro Martins Carrera, Laura Maria Cesar Schiesari, Dante Dianezi Gambardella, Adriano Massuda

**Affiliations:** a São Paulo School of Business Administration, Fundação Getulio Vargas, Avenida Dr Arnaldo 715, São Paulo, SP CEP-01246-904, Brazil; b School of Public Health – University of São Paulo, São Paulo, SP, Brazil; c Harvard T.H. Chan School of Public Health, Boston, MA, USA; d Real e Benemérita Associação Portuguesa de Beneficência, São Paulo, SP, Brazil

## Abstract

**Background:**

As of December 31, 2020, Brazil had the second-highest burden of COVID-19 worldwide. Given the absence of federal government coordination, it was up to the local governments to maintain healthcare provision for non-COVID health issues. In this descriptive study, we aimed to discuss the SUS functionality and resilience, describing the impact of the pandemic on non-COVID health services delivery while considering the regional inequalities of the allocation of financing health system, health infrastructure and health workforce.

**Methods:**

We used input-output framework based on the World Health Organization (WHO) Health System Building Blocks to estimate health system functionality and resilience. An ecological assessment was designed to calculated mean relative changes to compare the first year of the pandemic in Brazil with the previous one. All data used in this study were anonymized and made available by the Brazilian Ministry of Health. Input indicators were categorized in health system financing (federal funding received as well as expenditure of both state and city governments), health system's infrastructure (hospital beds) and health workforce (healthcare workers positions). Output indicators were categorized into nine different groups of service delivery procedures. To explore the relationship between the variation in procedures with socioeconomic conditions, we used the Socioeconomic Vulnerability Index (SVI).

**Findings:**

State governments had a 38·6% increase in federal transfers, while municipal governments had a 33·9% increase. The increase of ICU beds reached its peak in the third quarter of 2020, averaging 72·1% by the end of the year. The country also saw an increase in jobs for registered nurses (13·6%), nurse assistants (8·5%), physiotherapists (7·9%), and medical doctors (4·9%). All procedures underwent expressive reduction: Screenings (−42·6%); Diagnostic procedures (−28·9%); Physician appointments (−42·5%); Low and medium complexity surgeries (−59·7%); High complexity surgeries (−27·9%); Transplants (−44·7%); Treatments and clinical procedures due to injuries of external causes (−19·1%); Irrepressible procedures (−8·5%); and Childbirths (−12·6%). The most significant drop in procedures happened in the first quarter of the pandemic, followed by progressive increase; most regions had not yet recovered by the end of 2020. State-level changes in numbers of procedures point towards a negative trend with SVI.

**Interpretation:**

The Brazilian Government did not consider that socioeconomically vulnerable states were at a higher risk of being impacted by the overburden of the health system caused by the COVID-19, which resulted in poorer health system functionality for those vulnerable states. The lack of proper planning to improve health system resilience resulted in the decrease of a quarter of the amount of healthcare procedures increasing the already existing health disparities in the country.

**Funding:**

MCTIC/CNPQ/FNDCT/MS/SCTIE/DECIT N^o^ 07/2020.

**Research in context**

**Evidence before this study**

The response and impact of COVID-19 in the health system are widely discussed in the Brazilian media. However, few quantitative studies have systematically assessed these topics. They usually focus on a specific procedure or groups of procedures performed in a particular disease or condition. We used the search terms “Brazil,” “COVID-19,” “response,” “health system impact," “inequalities,” “procedures,” and synonyms to search Google Scholar and PubMed up to March 1, 2021, to identify relevant studies published in English or Portuguese. The few studies that approached the topic have focused on a limited number of procedures or specific regions and populations.

**Added value of this study**

Our study provides novel evidence on the possible determinants of the impact of the COVID-19 pandemic on the Brazilian health system while discussing it through the lenses of health system resilience and geographical inequalities. We use an extensive set of micro-level data from official governmental sources. This study shed light on the wildly speculated subject that is the performance of the public health system during the first year of the COVID-19 pandemic. We identify patterns relevant to policy design and epidemiological assessments in Brazil and elsewhere. Most notably, we found that the acute shock brought to the Brazilian public health system by the COVID-19 epidemic resulted in an acute drop in non-covid healthcare procedures in the country despite the increase in human, physical, and financial resources. We also show that the distribution of resources did not prioritize the most vulnerable states, which were the most affected by the drop in procedures.

**Implications of all the available evidence**

Similar discussions and concerns are starting to emerge in other countries, especially in Low- and Middle-Income Countries. Delays in diagnosis and treatment burden the historically underfunded health systems of countries with vast inequalities. Socioeconomically vulnerable populations face a greater risk of being impacted by this hidden wave. Policymakers should be aware that this scenario will continue to happen as COVID-19 is a major threat to health system and populations. Thus, allocating resources in socioeconomically vulnerable regions is imperative to reduce avoidable deaths in the near future.

Alt-text: Unlabelled box

## Introduction

Registering more than 36·55 cases and 0·93 deaths per thousand inhabitants as of December 31, 2020, Brazil had the second-highest burden of coronavirus disease 2019 (COVID-19) worldwide. In 2021, as of October 1st, the country recorded more 402,220 deaths caused by COVID-19, the highest number in the world. Federal government omissions in the management of the crises, obstruction of measures to control the spread of the virus, and usage of fake news to discredit regional and local health authorities were described as critical to the disastrous impact of COVID-19 in Brazil.^1^ On the other hand, state and municipal governments have taken a leading role in the health system response to the pandemic at the local level under highly unequal conditions.^2^

With a population of 211 million in 2019, Brazil has the 6^th^ largest population globally. Its 8·5 million km^2^ is divided into five regions (North, Northeast, Southeast, South, and Center-West), 27 Federative Units (UF) comprised of 26 states and the country's capital city, Brasília, and 5570 municipalities. The country's health system, Sistema Unico de Saúde (SUS), was founded after the 1988 Constitution approved its organizational principles - universality, comprehensiveness, and social participation – following the guidelines defined by the Brazilian sanitary movement. The SUS is the primary source of care for 75% of the population (Table 1).^3^ Since its beginnings, health system management has been decentralized to municipal governments.^4^ Health Regions were later created to group neighboring municipalities to integrate and organize the planning and the proper rendering of health actions and services.^5^

**Table 1:** Demographic and social indicators (2019) and epidemiological indicators on COVID-19 (2020).

					COVID-19	
REGION	Pop*	Size	SUS dependent*	Gini*	Cases	Deaths
	(/ 1M)	(/100,000 km^2^)	(%)		(/1000)	(/1000)
North	18.43	38.54	89.6	0.537	46.64	0.98
Northeast	57.07	15.58	87.8	0.559	33.26	0.84
Southeast	88.37	9.25	64.9	0.527	30.42	1.01
South	29.98	5.76	75.4	0.467	45.33	0.74
Center-West	16.30	16.12	78.0	0.513	53.75	1.10
Brazil	210.15	85.25	75.8	0.543	36.55	0.93

*Data from 2019

Despite its continental dimensions and high socioeconomic inequalities, Brazil's universal and decentralized system and previous well-successful experiences with other public health emergencies could have been advantageous for a more resilient response to the pandemic.^6^ The concept of resilience is used in studies of health systems to analyze the capacity to absorb the impacts of external shocks caused by epidemics, natural disasters, economic crises, or other causes without altering its operations and avoiding an increase of unmet health needs for different reasons.^7^ During the COVID-19 event, health systems resilience is used to benchmark country responses to the pandemic, offering important lessons for strengthening health systems.^8^

During previous epidemics in Brazil, such as those caused by Influenza (2009) and Zika (2015), health system interventions changed the course of the outbreaks. National coordination of the SUS led by the Ministry of Health (MoH) and local interventions implemented by the public health staff and Primary Health Care (PHC) teams linked to municipal governments were crucial for the responses.^9^^,^^10^ However, since 2015, SUS's capacity has been weakened by economic and political factors. The 2014 financial crisis was followed by long-term fiscal austerity policies implemented in 2016, aggravating the chronic public health underfunding and undermining the health system's reach.^11^ Furthermore, Bolsonaro's government replaced MoH experts with military personnel without prior public health expertise, causing significant damage to the SUS's national coordination.^12^

Given the absence of federal government coordination, state and municipal managers had to build strategies to face the pandemic. It was up to the local governments to maintain healthcare provision for non-COVID health issues. Examining Brazil's and Mexico's response to COVID-19 Knaul et al introduced the concept of “Punt Politics” when national leaders in federal systems defer or deflect responsibility for health systems decision-making to sub-national entities without evidence or coordination, reducing health system functionality and contributing to excess mortality.^13^ In this ecological study, we aimed to discuss the SUS functionality and resilience by exploring the pandemic's impact on non-COVID health procedures. We first described changes in input indicators of health system financing, infrastructure and workforce and then analyzed output indicators of healthcare utilization during 2020 compared to the previous year. We also explored the relationship between the variation in number of procedures and socioeconomic conditions.

## Methods

We used an input-output framework based on the World Health Organization (WHO) Health System Building Blocks elements to estimate health system functionality and resilience.^14^ We designed an ecological assessment that compares the quarters of the first year of the pandemic in Brazil with the respective quarters of the previous year. This decision was taken to account for seasonal variations within the service provision data. We opted not to use other approaches such as the mean values of previous years because we believe that the continuous decline of healthcare production since the economic crises of 2016 would inflate the estimates, not reflecting the scenario that SUS was facing immediately before the pandemic. Throughout the text, we often refer to 2019 as the “pre-pandemic” period and 2020 as the “pandemic” period. All data presented in this study are anonymized and made available by the Brazilian MoH through its open data repository (https://datasus.saude.gov.br/) and were downloaded in June 2021.

### Input-indicators

Health financing: We gathered information on governmental fund transfers from the Information System on Public Health Budgets (SIOPS). The SIOPS dataset contains all Federal funds transfers to state and municipal governments and their expenditures.

Health infrastructure: We used the Brazilian National Registry of Health Facilities (CNES) to obtain data on hospital beds and healthcare workers. We divided hospital beds into two groups, Intensive Care Unit (ICU) hospital beds and other hospital beds.

Health workforce: We extracted the mean values of medical doctors, registered nurses, nursing assistants, and physiotherapists for the period. Healthcare workers were grouped using the Brazilian Classification of Occupations (CBO) identification numbers. The CNES database enables the identification of workers through their National Health Registry number, which allows to estimate the total of individuals employed and on contract, thus permitting to estimate the average number of job positions occupied by individuals. We did not filter the workers data by their role in the establishment.

### Output-indicators

Health service delivery: Data on procedures are available monthly through the Outpatient Information System (SIA) and the Hospital Information System (SIH). The data used here contain the most up to date information as of June 2021, in order to avoid the usual 16-week delay in reporting. Once registered on the Brazilian health system, every procedure has an identification number. The health facility that performs the procedure needs to register details about it and provide information on the patients and their health conditions, using standardized forms, to receive payment for the procedure performed. The registration quality of some groups of procedures may vary according to financial incentives and convenience.

We created nine groups of procedures: diagnostic (diagnostical procedures such as radiology, magnetic resonance, or tomography), screening (cytopathological investigations, ultrasonography, endoscopies, and radiological interventions), irrepressible (oncological and nephrological treatments), physician appointments (all appointments with physicians and clinical treatments), low and medium complexity surgeries (all surgeries that did not require hospital admissions), high complexity surgeries (surgeries that required hospital admissions), transplants, external causes (treatments of injuries and accidents), and childbirths. A more thorough explanation of how the groups were created can be found on Supplementary Table 1. We excluded all COVID-related procedures from our analysis in order to focus on the impact on other conditions. We did this by explicitly excluding COVID-19 procedures (Supplementary Table 1) and excluding procedures that listed the ICD10 codes U071, U072, B342 or U04 as the primary diagnosis.

### Socioeconomic vulnerability index

To explore the relationship between the variation in procedures with socioeconomic conditions, we used the Socioeconomic Vulnerability Index (SVI) created by Rocha et al.^15^ The SVI ranges from 0 (least vulnerable) to 1 (most vulnerable) and was created by applying state-level socioeconomic indicators to a principal component analysis model.^15^

The mentioned datasets contain standardized geolocation codes for all the observations. The total number of procedures was obtained by calculating the sum of procedures conducted during each period; we used averages to assess the numbers of hospital beds and health workers. We aggregated procedures according to the municipality where the patient lived; when this information was not available, we used the information regarding the city where the procedure was performed. Lastly, to create the figures, we aggregated all variables at the Regional level. We aggregated all variables at the state and country levels to create the tables.

Municipal population estimates by municipality and year provided by the Brazilian Institute of Geography and Statistics (IBGE) were aggregated at the Health Region level. Monthly population estimates were calculated using linear interpolation, and rates per 10·000 inhabitants were calculated for all indicators and Health Regions. We used the relative change in percentages to estimate the increase or decrease of our indicators. All estimates and figures were generated using Stata v15·1.

### Role of the funding source

The funding agency had no role in data collection, analysis, interpretation, or writing this manuscript.

## Results

### Health system financing

In 2020, the proportional increase in federal transfers to states was 5% higher than the increase in federal transfers to municipalities. Collectively, the total received by municipalities was almost three times the amount received by the states. Only the North region had states (Acre and Amapá) where the state administration received fewer funds than the municipal administration of the same state. State administrations received a total of 29·8 billion reais in 2020, a 38·6% increase from 2019. Meanwhile, municipal administrations received 85·3 billion, which represents a 33·9% increase. States increased at least 11% (Roraima), and the states where municipalities experienced the lowest proportional increase was Rio de Janeiro, with only a 19% increase. The cities on the State of Minas Gerais received nine times what was given to the state (Table 2).

**Table 2:** Federal transfers and expenditure made by the administrative sphere's own resources in 2020 and relative change in relation to 2019.

	STATE	Federal Transfer			Expenditure		
		State	Mun.	Tot	State	Mun	Tot.
		/R$100M (R.C.%)	/ R$100M (R.C.%)	/ R$100M (R.C.%)	/ R$100M (R.C.%)	/ R$100M (R.C.%)	/ R$100M (R.C.%)
NORTH	Rondônia	3·93 (35·64)	6·15 (36·08)	10·08 (35·90)	9·11 (6·66)	6·61 (7·14)	15·72 (6·90)
	Acre	3·79 (47·68)	2·29 (28·11)	6·08 (39·64)	6·83 (5·81)	1·89 (5·84)	8·73 (5·85)
	Amazonas	7·25 (50·77)	12·58 (49·16)	19·83 (49·74)	24·86 (30·21)	13·44 (13·95)	38·31 (23·83)
	Roraima	2·05 (11·41)	2·13 (30·56)	4·18 (20·40)	5·69 (−7·67)	2·04 (10·13)	7·73 (−2·75)
	Pará	9·88 (88·67)	29·72 (29·79)	39·60 (40·75)	28·06 (15·81)	22·72 (10·70)	50·78 (13·50)
	Amapá	2·92 (44·94)	2·69 (73·49)	5·61 (57·34)	8·36 (40·90)	1·44 (0·28)	9·80 (33·08)
	Tocantins	5·21 (46·58)	7·32 (26·48)	12·52 (34·12)	11·85 (−3·53)	5·92 (12·85)	17·77 (1·40)
NORTHEAST	Maranhão	7·00 (70·57)	32·65 (18·36)	39·65 (25·12)	20·24 (3·40)	17·54 (11·22)	37·78 (6·33)
	Piauí	4·35 (23·63)	20·74 (38·72)	25·09 (35·85)	13·53 (33·38)	12·98 (11·00)	26·51 (21·09)
	Ceará	10·52 (49·02)	46·32 (33·69)	56·84 (36·29)	30·95 (18·01)	30·22 (6·42)	61·18 (12·00)
	Rio Grande do Norte	4·98 (51·98)	16·77 (41·47)	21·75 (43·75)	13·37 (16·84)	14·48 (10·98)	27·85 (13·74)
	Paraíba	3·47 (81·92)	24·01 (34·05)	27·49 (38·66)	12·18 (1·56)	12·49 (6·73)	24·67 (4·13)
	Pernambuco	22·52 (36·81)	34·23 (29·17)	56·75 (32·10)	36·66 (9·84)	27·94 (5·95)	64·60 (8·14)
	Alagoas	3·80 (65·10)	18·82 (21·06)	22·61 (26·73)	10·51 (4·46)	9·08 (2·62)	19·59 (3·61)
	Sergipe	5·42 (49·13)	9·17 (34·16)	14·60 (39·36)	8·98 (−0·42)	7·28 (6·89)	16·26 (2·74)
	Bahia	23·08 (30·80)	59·06 (33·70)	82·15 (32·87)	41·42 (1·95)	47·23 (8·33)	88·65 (5·28)
SOUTHEAST	Minas Gerais	12·48 (32·38)	116·46 (36·95)	128·93 (36·49)	66·07 (−2·25)	108·50 (8·49)	174·57 (4·19)
	Espírito Santo	10·21 (26·57)	11·20 (54·03)	21·41 (39·59)	19·58 (−3·17)	15·74 (2·29)	35·32 (−0·78)
	Rio de Janeiro	11·55 (57·82)	69·16 (19·44)	80·71 (23·75)	51·91 (3·20)	70·44 (−6·00)	122·35 (−2·28)
	São Paulo	73·26 (33·67)	138·95 (38·33)	212·21 (36·68)	188·54 (4·48)	323·42 (6·85)	511·96 (6·00)
SOUTH	Paraná	18·42 (21·53)	46·63 (39·91)	65·05 (34·17)	41·90 (5·39)	57·34 (−2·93)	99·24 (0·44)
	Santa Catarina	9·33 (29·29)	32·84 (34·91)	42·17 (33·62)	33·06 (13·71)	40·39 (1·28)	73·45 (6·56)
	Rio Grande do Sul	18·16 (54·60)	49·08 (44·03)	67·24 (46·74)	42·99 (0·93)	53·85 (1·05)	96·84 (1·02)
CENTER-WEST	Mato Grosso do Sul	3·30 (63·23)	15·26 (37·11)	18·57 (41·13)	15·65 (13·91)	17·39 (1·06)	33·04 (6·80)
	Mato Grosso	4·09 (27·48)	15·66 (27·80)	19·74 (27·73)	17·54 (10·84)	21·56 (12·40)	39·10 (11·74)
	Goiás	5·11 (34·29)	33·78 (35·34)	38·89 (35·21)	24·77 (5·33)	28·28 (7·11)	53·05 (6·32)
	Distrito Federal	12·13 (24·83)	-	-	-	-	15·72 (6·90)
	BRAZIL	298·22 (38·58)	853·67 (33·89)	1151·9 (35·07)	784·64 (8·34)	970·21 (5·79)	1754·85 (5·66)

**Figure 1:**
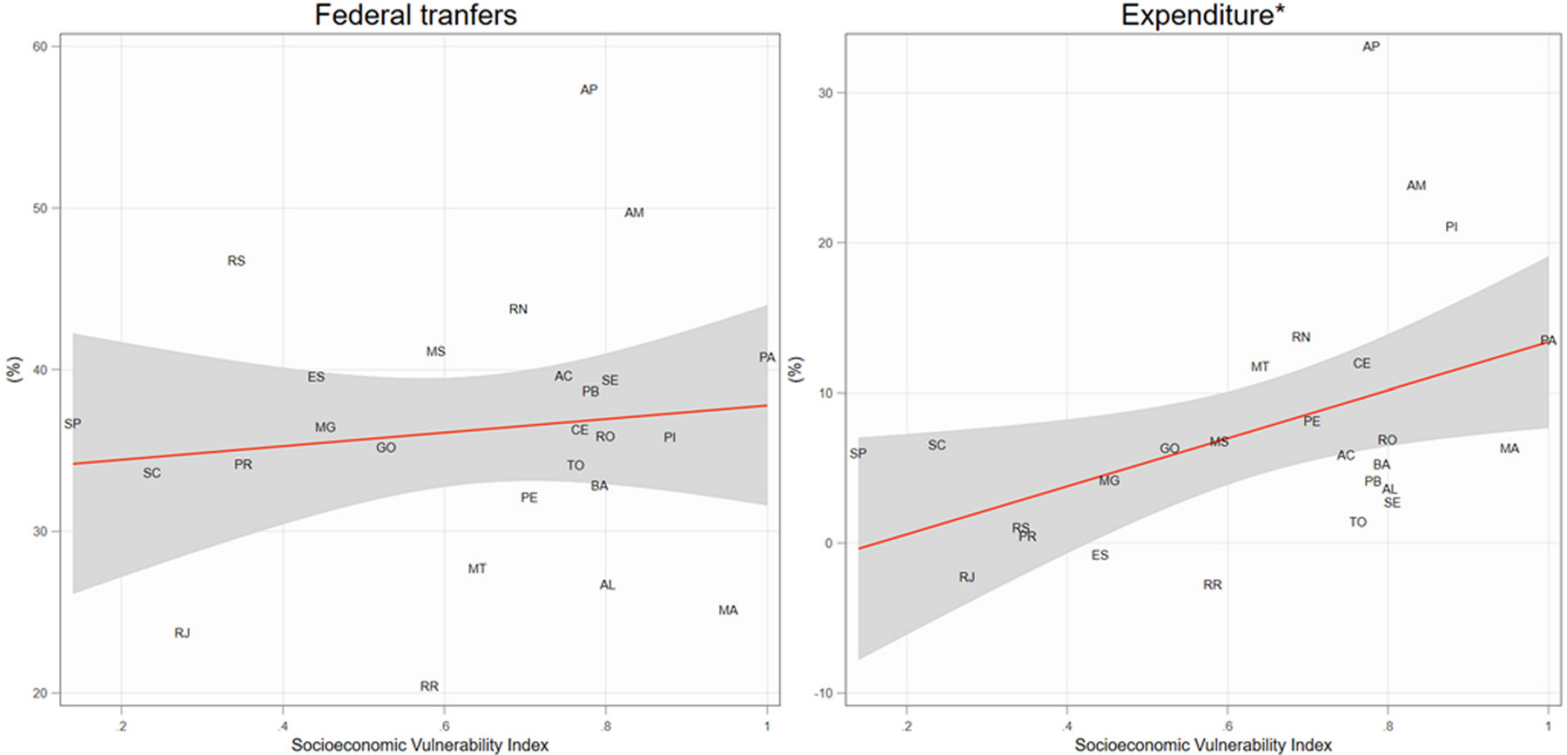
Correlations between Socioeconomic Vulnerability Index and relative change of federal transfers and expenditure with own resource by UF. *Significant at the 5% level.

Disparities are also reflected in the expenditure profile of governmental spheres. States spent less than municipalities, but their expenditures were almost 3% higher. The state administration of Roraima, Sergipe, Minas Gerais, and Espirito Santo spent less on health in 2020 than they did in 2019, even though their budget increased. The expenditure of the municipal administration increased in almost all states, with the only exception of Rio de Janeiro and Paraná. The proportional increase in expenses was modest compared with the increase in federal transfers (Table 2). When combined, we see that the federal transfers were somewhat egalitarian among federative units. However, their expenditure was not. The increase in spending was higher in more vulnerable regions (Figure 1).

### Health system infrastructure

**Figure 2:**
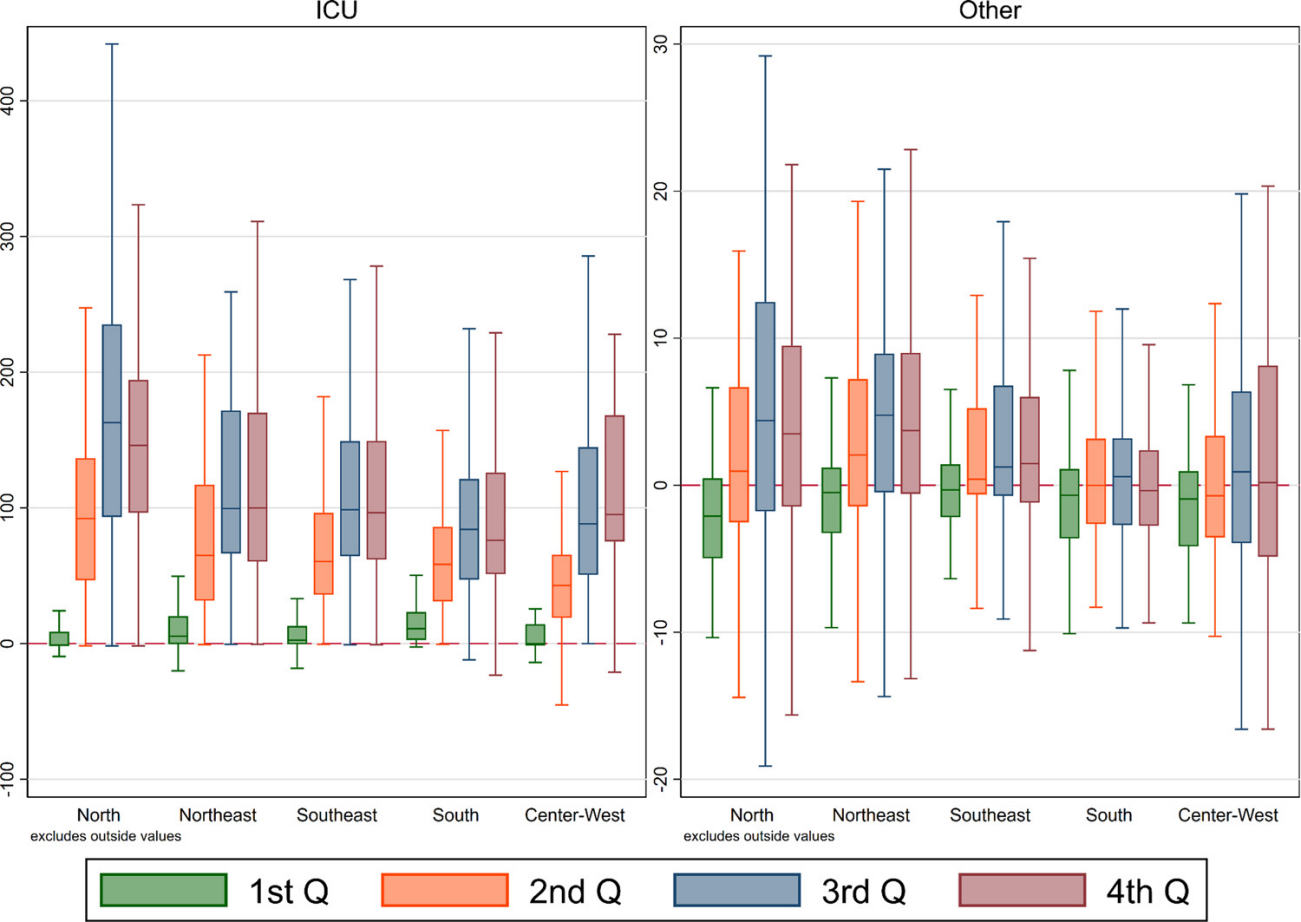
Relative change distribution of hospital beds rates in health regions by major region and quarter.

The increase of ICU beds reached its peak in the third quarter (Figure 2), averaging an increase of 72·1% by the end of 2020· The state that experienced the lowest growth was Rio de Janeiro, increasing by only 45·3% (Table 3). The North Region experienced the most significant mean increase in ICU beds. At the same time, the Center-West was the only region that, on average, maintained its rate of increase of ICU beds during the fourth quarter. The large interquartile ranges in the third quarter of the North region point to an unequal distribution of resources among its Health Regions (Figure 2). The increase of other hospital beds was moderate, and some Health Regions experienced a reduction in those resources.

**Table 3:** Rates of human and physical resources (per 1000 inhab.) in 2020 and its mean relative change (%) in relation to 2019.

	STATE	Hospital Beds		Health Workers			
		ICU	Other	Nurses	Nursing Assistants	Physiotherapists	Medical Doctors
NORTH	Rondônia	0·25 (83·7)	2·67 (0·6)	1·72 (22·7)	4·75 (16·6)	0·50 (23·5)	3·55 (9·0)
	Acre	0·17 (232·0)	1·97 (7·4)	1·58 (8·0)	3·85 (10·3)	0·34 (19·0)	2·84 (8·0)
	Amazonas	0·14 (102·9)	1·58 (3·0)	1·51 (19·7)	4·11 (13·0)	0·23 (16·8)	3·41 (14·6)
	Roraima	0·12 (185·7)	2·50 (29·3)	1·99 (18·6)	6·38 (9·4)	0·45 (13·5)	3·57 (16·0)
	Pará	0·15 (97·4)	1·87 (5·0)	1·03 (14·4)	2·65 (10·8)	0·25 (2·5)	2·02 (0·7)
	Amapá	0·13 (144·2)	1·60 (11·6)	1·52 (15·8)	5·09 (5·9)	0·51 (9·2)	2·05 (6·0)
	Tocantins	0·19 (136·3)	2·07 (−2·3)	2·30 (11·5)	5·29 (9·2)	0·58 (6·9)	3·33 (6·9)
NORTHEAST	Maranhão	0·14 (79·7)	2·18 (7·3)	1·42 (12·5)	3·30 (6·6)	0·32 (16·1)	1·83 (7·6)
	Piauí	0·18 (147·9)	2·55 (6·3)	1·62 (16·9)	3·91 (9·9)	0·60 (0·0)	2·72 (−0·4)
	Ceará	0·18 (101·1)	2·28 (6·5)	1·60 (18·2)	2·78 (13·9)	0·48 (15·1)	3·46 (10·2)
	Rio Grande do Norte	0·24 (99·2)	2·30 (5·6)	1·47 (12·9)	3·80 (8·0)	0·43 (8·3)	4·06 (6·6)
	Paraíba	0·18 (60·9)	2·26 (3·6)	1·93 (7·3)	3·23 (5·0)	0·63 (5·9)	3·68 (6·6)
	Pernambuco	0·28 (82·1)	2·53 (8·1)	1·69 (14·2)	4·20 (9·5)	0·46 (9·8)	3·92 (1·1)
	Alagoas	0·16 (83·0)	2·09 (13·3)	1·44 (18·3)	3·42 (8·6)	0·59 (13·2)	3·94 (9·3)
	Sergipe	0·20 (95·1)	1·62 (10·1)	1·41 (16·5)	3·91 (8·1)	0·46 (6·8)	5·27 (3·5)
	Bahia	0·19 (91·9)	2·11 (2·8)	1·72 (14·5)	3·50 (9·9)	0·54 (12·0)	4·15 (7·3)
SOUTHEAST	Minas Gerais	0·25 (75·7)	2·14 (4·7)	1·66 (13·0)	4·48 (8·7)	0·69 (6·9)	8·34 (6·8)
	Espírito Santo	0·36 (86·8)	2·20 (−1·1)	1·77 (14·9)	4·46 (10·3)	0·57 (0·9)	5·87 (−0·9)
	Rio de Janeiro	0·38 (45·3)	2·30 (−2·2)	1·92 (11·3)	4·58 (4·7)	0·59 (−0·3)	5·14 (−4·3)
	São Paulo	0·30 (67·8)	2·22 (2·8)	1·83 (12·1)	4·98 (7·2)	0·57 (8·6)	7·71 (3·6)
SOUTH	Paraná	0·26 (47·1)	2·58 (1·4)	1·67 (14·4)	4·05 (10·1)	0·64 (6·5)	7·48 (9·5)
	Santa Catarina	0·23 (101·7)	2·23 (−1·2)	1·80 (20·1)	4·26 (13·6)	0·66 (5·9)	8·48 (7·4)
	Rio Grande do Sul	0·23 (66·0)	2·79 (−0·4)	1·84 (11·9)	5·01 (7·9)	0·62 (5·3)	7·63 (4·9)
CENTER-WEST	Mato Grosso do Sul	0·23 (80·8)	2·19 (1·9)	1·80 (8·6)	4·12 (8·1)	0·57 (5·6)	6·20 (3·5)
	Mato Grosso	0·28 (65·5)	2·22 (−1·2)	1·65 (19·8)	3·83 (11·4)	0·52 (17·3)	4·37 (8·2)
	Goiás	0·24 (65·1)	2·67 (3·6)	1·34 (13·1)	3·29 (7·4)	0·45 (9·2)	4·68 (6·5)
	Distrito Federal	0·54 (80·2)	2·76 (5·3)	2·55 (14·8)	6·98 (9·6)	0·89 (22·8)	7·94 (12·2)
	BRAZIL	0·25 (72·1)	2·27 (3·0)	1·71 (13·6)	4·24 (8·51)	0·55 (7·86)	5·81 (4·87)

### Health system workforce

**Figure 3:**
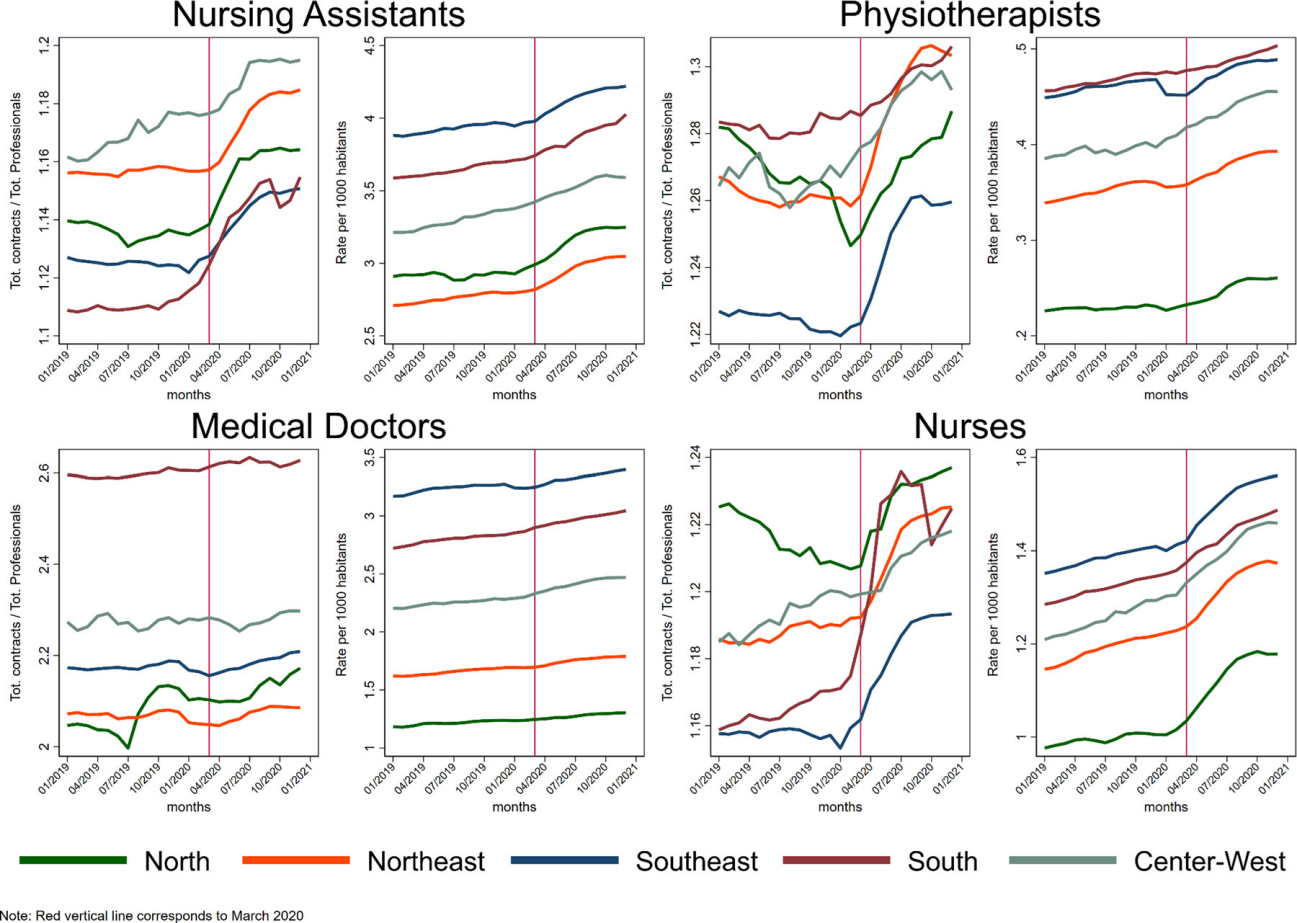
Trendlines of total of contracts signed divided by the total of workers and the rate per 1000 habitants of a given health profession group.

Parallel to the evident increase in ICU beds, although of a lower magnitude, was the increase in human resources jobs availability. The COVID-19 pandemic generated an increase in job positions of registered nurses (13·6%), nurse assistants (8·5%), physiotherapists (7·9%), and medical doctors (4·9%) in the country. The increase in the number of healthcare workers was minor. There is a continuous upward trend in all groups of health professions since 2018, with a slight increase after the first quarter of 2020 for nursing assistants, physiotherapists, and registered nurses. This effect is less evident in medical doctors (Figure 3). A decrease in healthcare jobs happened in only two states: Rio de Janeiro, which lost 0·3% of its physiotherapist positions and 4·3% of its medical doctors' jobs, and Piauí, which lost 0·4% of its medical doctors' positions (Table 3)

### Health system delivery

In 2020, Screenings (−42·6%); Diagnostic procedures (28·9%); Physician appointments (−42·5%); Low and medium complexity surgeries (59·7%); High complexity surgeries (−27·9%); Transplants (−44·7%); Treatments and clinical procedures due to injuries of external cause (−19·1%); Irrepressible procedures (-8·5%); Childbirths (−12·6%); and all other procedures (−15·5%) suffered a gradual reduction in all quarters. Collectively, the country saw a quarter reduction in its healthcare production (Table 4). A larger drop is seen in screenings, appointments with physicians, high complexity surgeries, transplants, low and medium complexity surgeries, and diagnostic procedures.

**Table 4:** Rates of procedures (per 100 hab.) in 2020 and its mean relative change (%) in relation to 2019.

	STATE	Screening	Irrepressible	Phys. Appoint.	Surgeries LMC	Surgeries HC	Transplants	Diagnostic	Ext. Causes	Childbirths	Other	Total
NORTH	Rondônia	24·6 (−61·0)	51·2 (−14·9)	2096·3 (−56·5)	24·3 (−93·8)	7·6 (−36·0)	0·0 (−55·0)	217·4 (−24·8)	1·9 (−1·6)	1·6 (−35·8)	2877·9 (−38·4)	5327·3 (−48·8)
	Acre	10·4 (−59·0)	15·8 (−7·0)	914·2 (−34·9)	13·5 (−88·7)	4·0 (−4·1)	0·0 (−30·8)	85·4 (−27·4)	0·3 (−3·0)	1·7 (−5·0)	1112·6 (−29·2)	2163·6 (−34·0)
	Amazonas	40·2 (−52·6)	29·3 (−0·8)	1819·6 (−40·5)	49·6 (−70·8)	7·6 (−35·0)	0·0 (−59·4)	171·1 (−32·3)	0·6 (−22·5)	4·7 (−23·8)	3172·0 (−27·8)	5334·6 (−34·1)
	Roraima	5·4 (−71·4)	6·8 (−34·9)	226·7 (−65·8)	3·8 (−58·2)	2·4 (−26·1)	0·0 (−60·0)	47·8 (−51·8)	0·3 (−34·8)	1·8 (−19·0)	391·9 (−53·6)	693·7 (−58·9)
	Pará	49·3 (−45·3)	52·6 (−3·4)	2767·0 (−46·4)	136·0 (−62·0)	15·9 (−24·9)	0·0 (−52·8)	394·0 (−12·8)	1·3 (−26·0)	5·3 (−23·1)	3035·4 (−35·9)	6739·9 (−39·8)
	Amapá	5·1 (−51·6)	12·4 (−7·4)	428·7 (−39·7)	6·3 (−19·9)	2·6 (−29·2)	0·0 (−57·1)	306·6 (330·5)	0·2 (−8·9)	2·0 (−13·8)	434·0 (−38·2)	1214·5 (−21·9)
	Tocantins	39·7 (−34·5)	40·1 (−11·3)	1985·8 (−41·3)	27·9 (−63·7)	9·3 (−33·5)	0·0 (−40·7)	163·3 (−41·9)	0·7 (−32·4)	3·1 (−24·2)	2854·4 (−31·4)	5146·0 (−36·3)
NORTHEAST	Maranhão	152·9 (−19·8)	93·7 (−11·7)	8864·6 (−36·9)	193·1 (−50·4)	23·7 (−29·1)	0·0 (−36·5)	597·1 (−24·8)	1·8 (−24·3)	8·3 (−22·9)	6312·5 (−32·6)	16397·6 (−35·0)
	Piauí	40·2 (−70·2)	96·0 (−6·2)	1506·2 (−47·2)	63·3 (−70·7)	12·3 (−41·1)	0·0 (−29·5)	124·8 (−57·9)	0·6 (−38·0)	3·8 (−19·3)	3672·0 (−38·0)	5685·6 (−43·1)
	Ceará	100·0 (−47·0)	165·3 (−6·5)	3916·6 (−49·7)	78·5 (−61·3)	31·1 (−20·3)	0·1 (−41·8)	312·8 (−55·7)	1·5 (−23·7)	7·2 (−19·4)	10647·4 (−14·9)	15407·9 (−29·7)
	Rio Grande do Norte	30·4 (−55·7)	66·3 (−5·3)	1690·1 (−57·5)	76·1 (−65·9)	11·7 (−25·5)	0·0 (−51·4)	121·4 (−37·6)	0·7 (−21·0)	2·4 (−14·6)	4440·5 (−9·5)	6491·3 (−32·2)
	Paraíba	72·2 (−52·6)	108·5 (−6·8)	2771·9 (−39·7)	150·3 (−18·8)	17·9 (−27·9)	0·0 (−62·5)	307·2 (−51·6)	0·6 (−10·9)	5·4 (−13·4)	7348·4 (−11·7)	10865·2 (−23·7)
	Pernambuco	72·7 (−52·5)	103·5 (−4·5)	2871·7 (−43·6)	89·8 (−39·4)	14·7 (−33·5)	0·0 (−61·1)	343·2 (−13·4)	0·8 (−22·0)	4·8 (−15·8)	5969·0 (−21·1)	9551·9 (−30·2)
	Alagoas	78·5 (−42·0)	71·6 (−8·3)	2024·3 (−40·5)	24·8 (−77·3)	12·8 (−31·2)	0·0 (−65·9)	165·2 (−28·9)	0·9 (12·3)	5·0 (−0·1)	4442·6 (−17·6)	7126·9 (−27·3)
	Sergipe	25·1 (−53·7)	38·4 (2·6)	1611·0 (−19·9)	21·5 (−67·8)	6·5 (−24·8)	0·0 (−73·1)	92·8 (−43·1)	0·2 (−1·6)	3·8 (3·1)	2930·4 (−25·8)	4765·0 (−25·3)
	Bahia	157·6 (−49·8)	189·0 (−4·6)	5491·0 (−37·4)	102·0 (−61·0)	35·8 (−28·9)	0·1 (−60·5)	479·9 (−32·6)	2·3 (−14·1)	12·0 (−10·4)	11776·4 (−15·6)	18573·7 (−25·3)
SOUTHEAST	Minas Gerais	471·3 (−42·7)	845·9 (−9·1)	19681·3 (−42·2)	436·6 (−75·6)	119·9 (−26·3)	0·4 (−42·7)	2153·1 (−32·2)	8·5 (−8·2)	26·5 (−9·8)	62741·0 (−9·5)	87212·8 (−21·9)
	Espírito Santo	9·6 (−57·9)	12·9 (−9·3)	422·8 (−54·5)	6·9 (−69·0)	2·8 (−30·9)	0·0 (−53·3)	51·6 (−42·4)	0·1 (−24·7)	0·6 (−6·5)	1794·7 (−19·6)	2310·9 (−30·7)
	Rio de Janeiro	53·3 (−43·3)	61·4 (−11·3)	2569·0 (−44·1)	46·3 (−63·1)	8·9 (−26·0)	0·0 (−40·7)	376·0 (−19·1)	0·6 (−1·1)	2·8 (−4·2)	4522·0 (−11·8)	7804·9 (−27·5)
	São Paulo	715·9 (−37·9)	477·2 (−7·3)	22167·2 (−39·4)	340·0 (−67·5)	82·1 (−28·6)	0·4 (−37·4)	2609·7 (−28·9)	3·6 (−25·3)	18·6 (−7·9)	69851·6 (−12·7)	96777·4 (−22·1)
SOUTH	Paraná	177·2 (−41·6)	167·5 (−11·5)	6286·9 (−39·8)	98·7 (−68·3)	38·2 (−30·2)	0·1 (−39·5)	541·2 (−33·8)	2·1 (−9·8)	6·3 (−14·3)	21413·6 (−13·0)	28967·6 (−22·1)
	Santa Catarina	153·6 (−35·6)	108·0 (−14·2)	5228·7 (−36·3)	93·9 (−66·2)	26·0 (−33·5)	0·1 (−49·1)	485·7 (−31·2)	0·9 (−31·1)	5·3 (−14·2)	16450·9 (−15·3)	22815·5 (−22·5)
	Rio Grande do Sul	273·1 (−34·1)	240·6 (−13·5)	7656·0 (−33·6)	234·5 (−62·4)	49·5 (−21·8)	0·1 (−47·3)	842·9 (−24·2)	1·8 (−18·1)	7·9 (−7·8)	17853·2 (−15·0)	27596·3 (−22·7)
CENTER−WEST	Mato Grosso do Sul	25·3 (−52·6)	32·3 (−4·5)	1047·6 (−46·3)	16·3 (−64·6)	5·9 (−29·3)	0·0 (−53·6)	76·6 (−56·3)	0·3 (−30·9)	1·4 (−10·4)	2479·8 (−27·0)	3709·5 (−35·2)
	Mato Grosso	66·8 (−49·3)	112·7 (−6·7)	4895·9 (−42·4)	718·0 (256·4)	20·2 (−28·7)	0·1 (−32·5)	463·0 (−30·0)	1·7 (−33·3)	5·9 (−15·5)	8331·9 (−30·8)	14735·2 (−32·9)
	Goiás	97·8 (−50·1)	138·8 (−11·5)	4715·4 (−41·1)	274·8 (30·6)	24·6 (−23·7)	0·1 (−31·3)	410·9 (−34·5)	1·5 (−36·0)	4·4 (−15·2)	11757·5 (−10·7)	17539·4 (−22·5)
	Distrito Federal	3·7 (−39·7)	5·6 (−3·1)	155·5 (−23·0)	1·8 (−52·3)	1·2 (−14·3)	0·0 (−55·6)	20·1 (−7·2)	0·1 (−18·3)	0·4 (−9·8)	675·0 (−15·5)	869·5 (−17·0)
	BRAZIL	3181·84 (−42·53)	3251·47 (−8·48)	120570·00 (−41·16)	2957·66 (−59·66)	587·78 (−27·89)	1·74 (−44·74)	13052·88 (−28·90)	33·98 (−19·12)	151·91 (−12·60)	306530·10 (−15·46)	454968·90 (−25·60)

**Figure 4:**
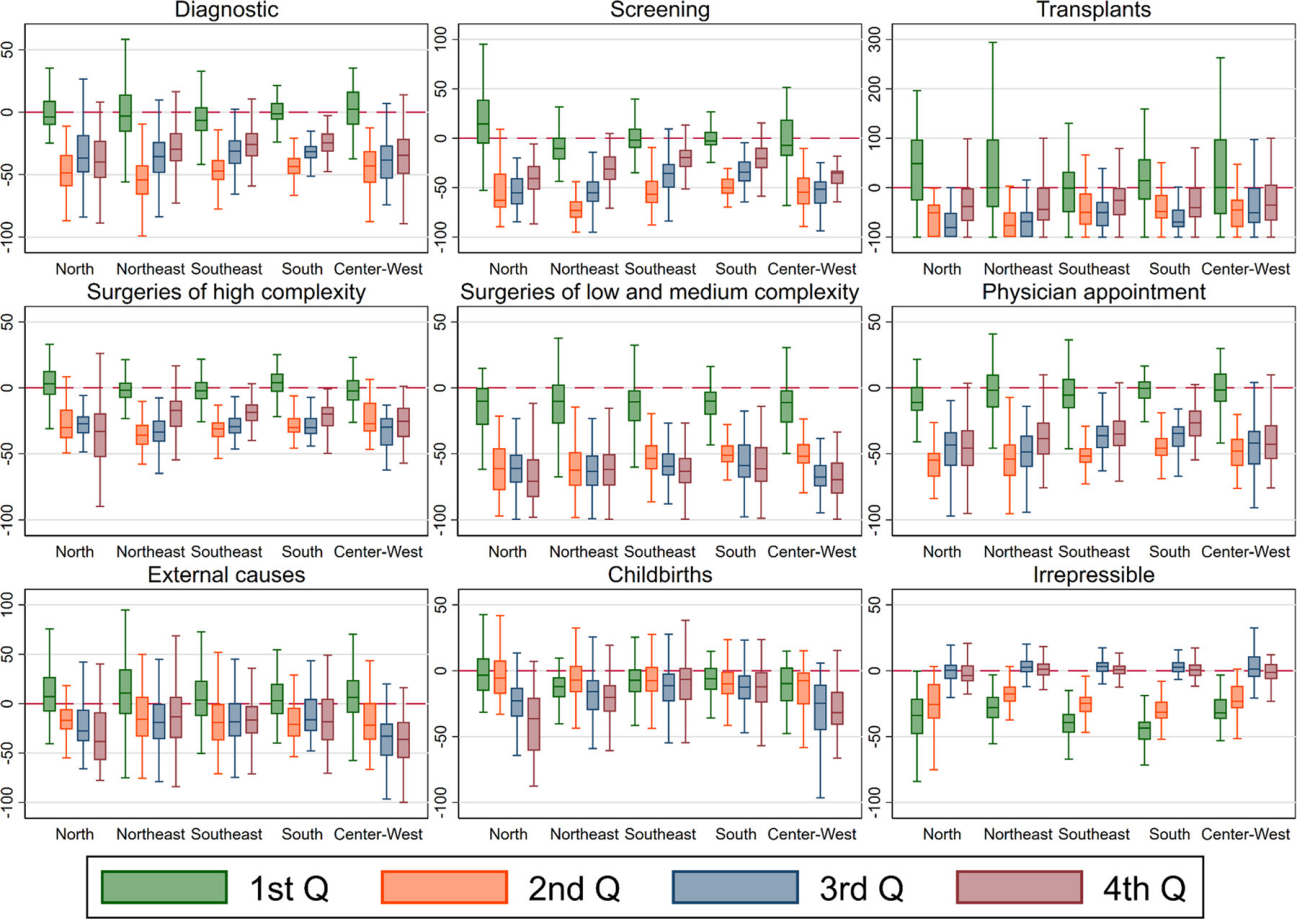
Relative change distribution of procedures rates in health regions by procedure group, major region, and quarter. *Significant at the 5% level.

Surgeries of low and medium complexity, transplants, and screening procedures were the most affected, with most states experiencing a reduction of more than 50% throughout 2020· Treatments and clinical procedures due to external cause injuries experienced a slow but steady decline in all regions. The same pattern is seen in childbirths, and irrepressible procedures were also affected, especially in the Northern region. The prevalent pattern throughout the year was one of an inverted "J," showing a strong contraction during the second quarter with a gradual recovery in the third and fourth quarters, but insufficient to go back to the pre-pandemic production. Low and medium-complexity surgeries were the exception, with almost no recovery achieved during the period (Figure 4).

**Figure 5:**
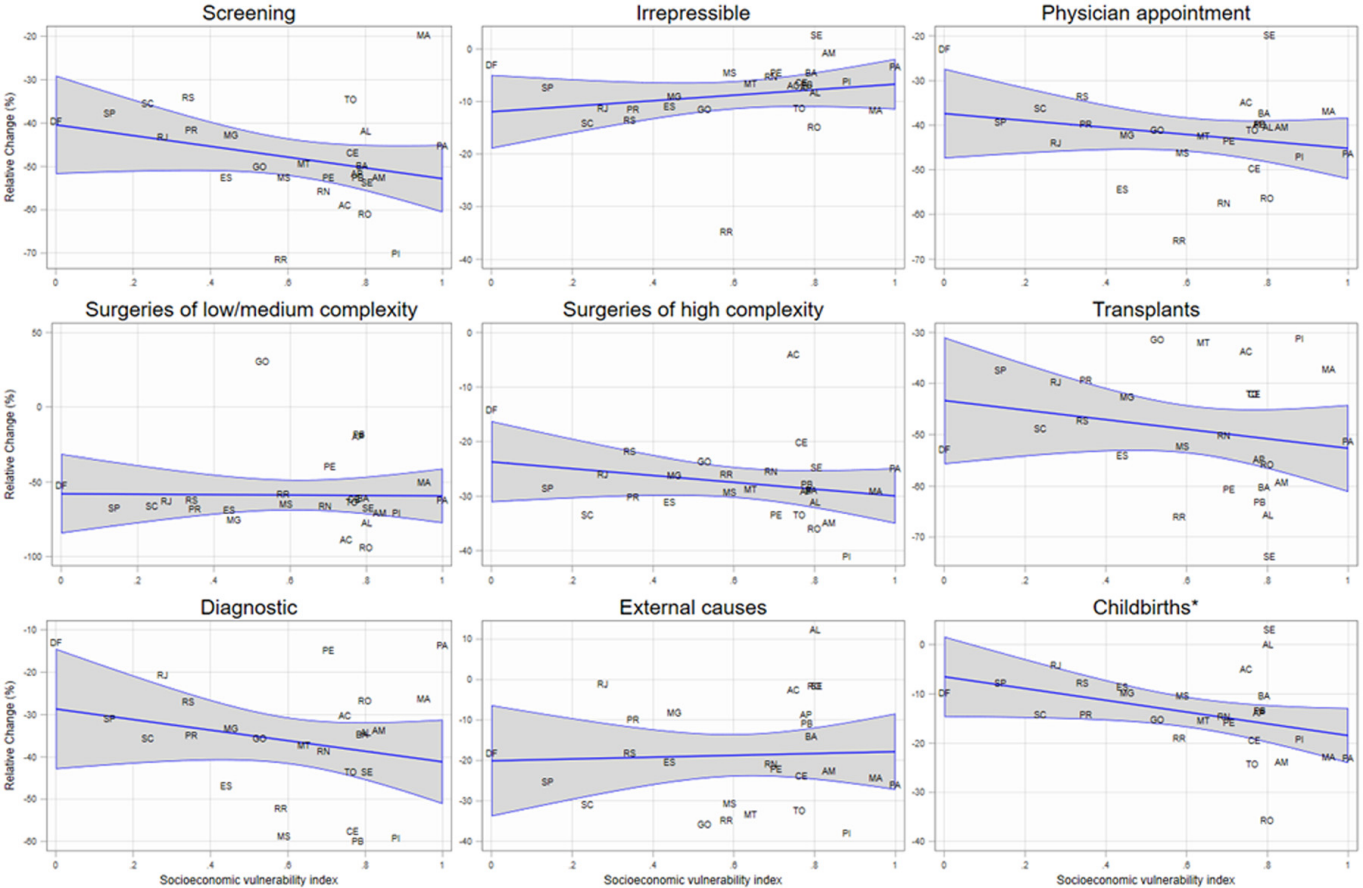
Correlations between Socioeconomic Vulnerability Index and relative change of healthcare production by procedure group and UF. *Significant at the 5% level.

**Figure 6:**
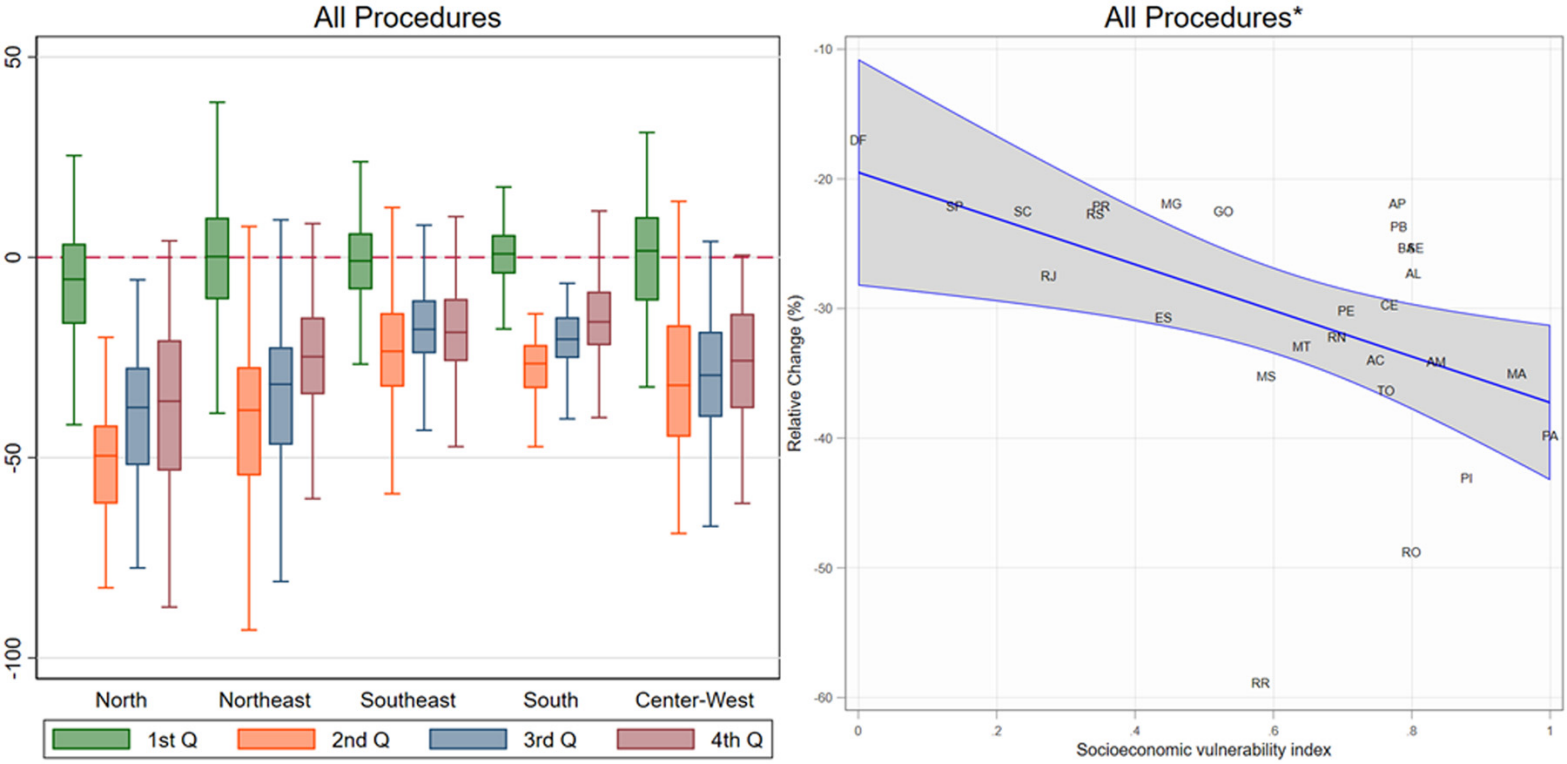
Relative change distribution of procedures rates in health regions by major region and quarter and correlation between Socioeconomic Vulnerability Index and relative change of healthcare production by UF. *Significant at the 5% level.

Scatter plots of state-level changes in numbers of procedures point towards a negative trend with SVI, meaning that more vulnerable states experienced a more considerable drop in screenings, physician appointments, surgeries of high complexity, transplants, childbirths, and diagnostic procedures. The impact in procedures and treatments for external causes and low and medium complexity surgeries doesn't appear to correlate with SVI. Irrepressible procedures were the only ones to indicate a positive trend in the relationship between the relative change of procedures and the SVI. The most significant drop in procedures happened in the first quarter of the pandemic (March-May 2020), followed by a progressive stabilization. However, most regions had not yet recovered by the end of 2020 (Figures 5 and 6).

## Discussion

Health systems worldwide are experiencing profound and prolonged shocks from the COVID-19 pandemic, raising attention to health system resilience. Haldane et al. described that while there is no one-size-fits-all path to resilience, well-performing countries have implemented similar strategies against COVID-19. Strategies such as including comprehensive and coordinated responses that consider and address health and well-being as being intertwined with social and economic factors; ensuring resources to maintain pandemic-related and non-related routine and acute care; and learning, monitoring and adjusting strategies according to evidence or the evolving epidemiological scenario were key in the responses.

In Brazil, however, Bolsonaro's government pushed the health system response towards the opposite direction. The acute shock caused by the COVID-19 resulted in a sharp drop in non-covid healthcare procedures in the SUS. Results show that the distribution of resources did not prioritize the most vulnerable states, which were the most affected by the drop in procedures. Thus, the increase in financing, workforce, and infrastructure was not allocated to prevent the decrease in diagnostic and screening procedures is particularly alarming, as well as the drop in treatments that require complex surgeries (i.e., oncology, neurology and cardiac surgery).

Preparedness is a crucial aspect of resiliency in health systems.^7^ Brazil has shown in the past the capacity to deal with shocks from disease outbreaks. The country dealt with Dengue, Zika, and Yellow Fever outbreaks by relying on collaboration among the three levels of government, epidemiological surveillance, primary health, and vaccination taskforces.^10^^,^^16^^,^^17^ However, in the past decade, the Brazilian health system was characterized by chronically insufficient financing,^18^ reductions in federal resources^19^ and fiscal austerity measures,^20^ which culminated in a systematic defunding of SUS.^21^ More recently, the discoordination of the national processes that rule federal financial transfers to states and municipalities also substantially impacted the pandemic response.

The discoordination reduces federal accountability, impairing the communication between governmental powers preventing a timely response.^22^^,^^23^ The discoordination of the health system had significant impacts in critical areas such as the distribution of personal protective equipment (PPE)^24^ oxygen, and mechanical ventilators to hospitals.^25^ Brazil passed its first provisional measure for the COVID-19 response on March 13th, 2020. During this initial phase, despite recognizing the need for a health system's expansion to deal with the pandemic,^26^ normative measures disallowed the use of federal transfers to increase human and physical resources. This restriction was only lifted in mid-April, explaining the increase in hospital beds from April to December (Figure 2).^27^

A health system's ability to maintain improvements caused by the response to shock is an essential element of resilience.^28^ Researchers recognized early on the pandemic that Brazil lacked hospital beds and ventilators to supply the incoming demand.^29^ Our results show that the country reached its peak in ICU beds supply in the second semester 2020. The North region experienced an especially accentuated increase, as a large portion of the area had a chronic shortage of ICU beds before 2020.^30^ Thus, it is particularly alarming that, in the 4th quarter, the North region had already lost some of the ICU beds acquired during the 3rd one. A small number of regions also lost other types of hospital beds; this was likely caused by repurposing those other hospital beds into ICU facilities.^31^ This repurposing likely contributed to the drop in healthcare procedures unrelated to COVID-19 treatment and control.

The significant increase of healthcare job positions but not in the number of healthcare workers probably imposed substantial influence on the number of front-line workers reporting burnout and experiencing a decline in mental health and overall wellbeing.^32^^,^^33^ Medical doctors did not experience a similar change in the rate per 1000 inhabitants or contracts per worker ratio. This is likely because doctors already had the highest proportion of job contracts per doctor. Thus, medical doctors are already in short supply in a health system that heavily relies on their work. Our graph in Figure 5 only shows trends starting in 2019, but Brazilian healthcare workers have been subjected – and sometimes succumbed to – overworking and burnout for quite some time.^34^ The dissolution of legislative measures that increased the legal limit of hours healthcare workers could work per week likely aggravated the already grim reality these workers were facing.^35^ It is necessary to further inquiry into the dynamics that caused the loss of job positions for medical doctors and physiotherapists in Rio de Janeiro e Piauí.

The impact of COVID-19 on health systems across the world was seen mainly as a problem to be dealt with in the present, with little planning regarding its future impacts. However, the near collapse of health systems and the fear caused by a scientific denialist government in Brazil affected the pandemic response and set a pessimistic perspective for the future. Procedures to care for non-covid related causes had an intense drop in 2020. A proportion of those who avoided going to hospitals or clinical appointments or had their visits cancelled by physicians due to an overcrowded hospital or fear of infection will lead to poorer outcomes in the near to mid future.^36^ Those individuals - especially those with chronic conditions - are referred to as “invisible patients,” The consequence is an incoming “third wave”, as it was called in 2021.^37^

The drop in diagnostic and screening procedures is particularly concerning. The sudden drop in the reporting of new cancer cases is worrisome.^38^ There is an indication that the pandemic already impacted patients potentially needing surgeries by delaying diagnostic and treatment.^39^ The impact in oral and oropharyngeal biopsies^40^ and hospitalizations^41^ have been documented in Brazil, but more studies are required to explore the implications in other cancer types. The same scenario seen in diagnostic and screening procedures is also found in surgeries, especially low and medium complexity ones. Social distancing and the recommendation that individuals did not leave their homes unless strictly necessary, together with hospitals postponing procedures to focus on COVID-19 patients, meant a reduction of elective surgeries. This fact did not occur only in Brazil. Countries with more robust health systems, such as Italy^42^ Spain^43^ and different regions of Asia, also reported an impact in elective procedures.^44^^,^^45^ Although part of the drop in procedures can be attributed to changes in the population's behavior, we argue that the lack of plans of action in place, such as screening campaigns adapted to the COVID-19 reality and strategies incentivizing vulnerable populations to go to hospitals in the case of any non-COVID-19 related symptoms, led to the reduction of timely diagnoses and procedures.

The WHO recommends social isolation to slow the rate of infection. Some Brazilian governors and mayors adopted the measure despite the federal government's reluctance. These measures also impacted injuries by external causes, reducing the number of hospitalizations that would have happened under normal circumstances. This study shows that all regions experienced a drop in medical procedures related to external causes in the first quarter of the pandemic, likely due to a decline in traffic and road accidents.^46^^,^^47^ The impact on deaths by external causes is also likely one of the main factors of the drop in organ transplantations.^48^

The drop in childbirths shown in this study is yet unexplored in Brazil. Our data are only related to childbirths performed at the public health system, but it is unlikely that women moved to the private sector in large quantities. The more likely explanation is that the economic impact of the COVID-19 pandemic and the fear of infection made women decide to postpone a possible pregnancy. A similar scenario was seen during the Zika Virus outbreak of 2016 in Brazil.^49^ There is no substantial evidence that this trend, which appears to have happened worldwide, should be attributed to sociocultural factors alone.^50^^,^^51^ The possibility that this drop should be attributed to physiopathological effects of the SARS-CoV-2 is being investigated; however, more studies are needed to explore causal links.^52^^,^^53^

Irrepressible procedures show a different pattern than the other groups. The decline in the number of procedures seems to have started before the pandemic arrived in the country, and then it slowly returned to its typical values. This is not an effect of the pandemic but rather the result of a change in the registry norms for some oncological treatment procedures, implemented in May 2019.^54^ The robust stabilization seen in the last two quarters of 2020 suggests that health facilities were able to maintain their levels of oncological and nephrological treatments.

Despite the country's health services inequalities, the evidence exposes a critical similarity of the country's response: Brazil's effort to deal with the pandemic was centered around the hospital's human and physical resources instead of preventive actions on primary care. Although further investigation is needed to infer causation from this policy decision, it is possible to argue that SUS and its decentralized structure rapidly increase health system capacity when increased financial support is given.

This aspect highlights a positive element of SUS resilience. Despite being worsened by the lack of federal support to manage the crises, health system functionality remains due to municipal government's role in service delivery, ensuring large-scale public health interventions in a continental and profoundly unequal country. As a result, more than 143 million Brazilians (70% of population) were fully vaccinated for COVID-19 as of January 2022,^55^ despite Bolsonaro's government rhetoric against vaccination.

Regarding the federal response in the COVID-19 pandemic, the federal law for Health Emergency of National Importance (ESPIN – *Emergência de Saúde Pública de Importância Nacional*) is clear in attributing an explicit mandate to the MoH for coordinating the health system responses. A Parliamentary Commission was established in 2021 to investigate the federal government's omissions in responding to the pandemic. The president was charged for nine crimes, and the Attorney General's Office opened six preliminary investigations to analyze facts reported in the Covid Parliamentary Commission report.

Our study has some limitations. All indicators presented were compared to a single year (2019), which may not reflect variability from the past period. This study also did not consider funding from congressmen amendments that could have impacted the funding profile and resource volume. The registry of healthcare procedures, health facilities characteristics, and health spending is prone to errors, delays, and biases. However, we believe that, although this limitation has some impact, its effect is probably attenuated by the data wrangling process of the datasets and by the decentralized nature of the data registration. It is also possible that the pandemic could have substantially changed the proportion of the population covered by private insurance. Although there was an increase in this ratio, it is unlikely to have impacted our results.^56^ Another limitation is the fact that this paper is mainly descriptive. We did not intend to draw causality on our work but instead to present the pandemic's impacts quantitatively and systematically.

Continuous defunding and the breach of the SUS collaborative management undermined the health system functionality and weakened the country's historical resilience to deal with novel pandemics. Although the federal government increased the funding to states and municipalities, it failed to properly distribute the funding and support the health system's response to those with higher needs. State and municipal governments mitigated the impact despite the continuous mismanagement by using their own resources to cover the additional expenditure required to increase infrastructure and health workers positions. However, these efforts had limitations and were not enough to completely stop the overburden of the health system caused by COVID-19, resulting in poorer outcomes. The lack of proper planning led to the 25% decrease in healthcare procedures and increased the already existing health disparities in the country. The repressed procedures of 2020 and 2021 will burden the country for the years to come. It is necessary to investigate the extent of this impact to better prepare the country to its consequences.

## Contributors

Alessandro B. and Adriano M. jointly conceived the study and developed the study outline. Alessandro B. was responsible for data curation and analysis and wrote the first draft with input from all authors. All authors contributed to the subsequent drafts and the final manuscript. All authors had access to all estimates presented in the paper and had final responsibility for the decision to submit for publication

## Data sharing statement

All data presented in this study are anonymized and made available by the Brazilian MoH through its open data repository (https://datasus.saude.gov.br/)

## Declaration of interests

The authors declare no conflict of interest

## Appendix Supplementary materials

Image, application 1

## References

[1] Ortega F., Orsini M. (2020). Governing COVID-19 without government in Brazil: ignorance, neoliberal authoritarianism, and the collapse of public health leadership. Glob Public Health.

[2] Gleriano J.S., Fabro G.C., Tomaz W.B., Goulart B.F., Chaves LD. (2020). Reflections on the management of Brazilian unified health system for the coordination in facing COVID-19. Esc Anna Nery.

[3] Viacava F., Oliveira R.A., Carvalho C.D., Laguardia J., Bellido JG. (2018). SUS: oferta, acesso e utilização de serviços de saúde nos últimos 30 anos. Ciênc. Saúde coletiva.

[4] Macinko J., Harris M.J. (2015). Phil D. Brazil's family health strategy—delivering community-based primary care in a universal health system. N Engl J Med.

[5] Viana A.L., Bousquat A., Pereira A.P. (2015). Typology of health regions: structural determinants of regionalization in BrazilI. Saúde Soc.

[6] Croda J., Oliveira W.K., Frutuoso R.L. (2020). COVID-19 in Brazil: advantages of a socialized unified health system and preparation to contain cases. Rev Soc Bras Med Trop.

[7] Thomas S, Sagan A, Larkin J, Cylus J, Figueras J, Karanikolos M. Strengthening health systems resilience: key concepts and strategies [Internet]. Copenhagen: World Health Organization; 2020 Jun [cited 2021 May 5]. Available from: https://europepmc.org/article/med/32716618#free-full-text 32716618

[8] Haldane V., De Foo C., Abdalla S.M. (2021). Health systems resilience in managing the COVID-19 pandemic: lessons from 28 countries. Nat Med.

[9] Domingues C.M., de Oliveira W.K., Brazilian Pandemic Influenza Vaccination Evaluation Team (2012). Uptake of pandemic influenza (H1N1)-2009 vaccines in Brazil, 2010. Vaccine.

[10] Castro MC. (2016). Zika virus and health systems in Brazil: from unknown to a menace. Health Syst Reform.

[11] Massuda A., Hone T., Leles F.A., de Castro M.C., Atun R. (2018). The Brazilian health system at crossroads: progress, crisis and resilience. BMJ Glob Health.

[12] Lancet T. (2020). COVID-19 in Brazil:“So what?”. Lancet.

[13] Knaul F.M., Touchton M., Arreola-Ornelas H. (2021). Punt politics as failure of health system stewardship: evidence from the COVID-19 pandemic response in Brazil and Mexico. Lancet Reg Health Am.

[14] World Health Organisation (2010).

[15] Rocha R., Atun R., Massuda A., Rache B, Spinola P, Nunes L (2021). Effect of socioeconomic inequalities and vulnerabilities on health-system preparedness and response to COVID-19 in Brazil: a comprehensive analysis. Lancet Glob Health.

[16] Salles T.S., da Encarnação Sá-Guimarães T., de Alvarenga E.S. (2018). History, epidemiology and diagnostics of dengue in the American and Brazilian contexts: a review. Parasites Vectors.

[17] Possas C., Lourenço-de-Oliveira R., Tauil P.L. (2018). Yellow fever outbreak in Brazil: the puzzle of rapid viral spread and challenges for immunisation. Mem Inst Oswaldo Cruz.

[18] Vieira FS. (2016). Impact of recent decisions and discussions on the Brazilian public health system financing. Saúde Debate.

[19] Lopes L.D., de Assis Acurcio F., Diniz S.D., Coelho T.L., Andrade EI. (2019). (Un) Equitable distribution of health resources and the judicialization of healthcare: 10 years of experience in Brazil. Int J Equity Health.

[20] de Souza LE. (2017). The right to health in Brazil: a constitutional guarantee threatened by fiscal austerity. J Public Health Policy.

[21] Santos L. (2018). The first 30 years of the SUS: an uncomfortable balance?. Cienc Saude Coletiva.

[22] Abrucio F.L., Grin E.J., Franzese C., Segatto C.I., Couto CG. (2020). Combating COVID-19 under Bolsonaro's federalism: a case of intergovernmental incoordination. Rev Adm Pública.

[23] de Almeida Lopes Fernandes G.A., Saturnino Pereira B.L. (2020). The challenges of funding the Brazilian health system in fighting the COVID-19 pandemic in the context of the federative pact. RAP Rev Bras Adm Pública.

[24] Martin-Delgado J., Viteri E., Mula A. (2020). Availability of personal protective equipment and diagnostic and treatment facilities for healthcare workers involved in COVID-19 care: a cross-sectional study in Brazil, Colombia, and Ecuador. PLoS One.

[25] Noronha K.V., Guedes G.R., Turra C.M. (2020). The COVID-19 pandemic in Brazil: analysis of supply and demand of hospital and ICU beds and mechanical ventilators under different scenarios. Cad Saúde Pública.

[26] World Health Organisation Joint Mission, Report of the WHO-China joint mission on coronavirus disease 2019 (COVID-19). https://www.who.int/docs/default-source/coronaviruse/who-china-joint-mission-on-covid-19-final-report.pdf (2020). Accessed 11 March 2021

[27] Fernandes G.A., Pereira BL. (2020). The challenges of funding the Brazilian health system in fighting the COVID-19 pandemic in the context of the federative pact. Rev Adm Pública.

[28] Abimbola S., Topp S.M. (2018). Adaptation with robustness: the case for clarity on the use of ‘resilience’ in health systems and global health. BMJ Global Health.

[29] Rache B, Rocha R, Nunes L, Spinola P, Malik AM, Massuda A. *Necessidades de Infraestrutura do SUS em Preparo à COVID-19: Leitos de UTI, Respiradores e Ocupação Hospitalar*. São Paulo: Instituto de Estudos para Políticas de Saúde; 2020. v1. http://www.epsjv.fiocruz.br/sites/default/files/files/NT3%20vFinal.pdf. Accessed 15 April 2020.

[30] Castro MC, de Carvalho LR, Chin T. (2020 Jan). Demand for hospitalization services for COVID-19 patients in Brazil. MedRxiv.

[31] Pedroso M.C., Pires J.T., Malik A.M., Pereira AJ. (2021). HCFMUSP: resiliência como resposta à pandemia de COVID-19. Rev Adm Contemp.

[32] Pinho R.D., Costa T.F., Silva N.M. (2021). Mental health and burnout syndrome among postgraduate students in medical and multidisciplinary residencies during the COVID-19 pandemic in Brazil: protocol for a prospective cohort study. JMIR Res Protoc.

[33] Ornell F., Halpern S.C., Kessler F.H., Narvaez JC. (2020). The impact of the COVID-19 pandemic on the mental health of healthcare professionals. Cad Saude Publica.

[34] Messenger JC, Vidal P. *The Organization of Working Time and Its Effects in the Health Services Sector: A Comparative Analysis of Brazil*. South Africa and the Republic of Korea: ILO; 2015. v1. http://www.oit.org/wcmsp5/groups/public/---ed_protect/---protrav/---travail/documents/publication/wcms_342363.pdf. Accessed 8 October 2020..

[35] Presidência da República, Secretaria-Geral. Provisional measure N° 927, March 22, 2020, http://www.planalto.gov.br/ccivil_03/_ato2019-2022/2020/mpv/mpv927.htm. Accessed 22 January 2020.

[36] How hospitals can meet the needs of non-Covid patients during the pandemic. https://hbr.org/2020/07/how-hospitals-can-meet-the-needs-of-non-covid-patients-during-the-pandemic. Accessed 29 August 2021.

[37] Mendes EV. O lado oculto de uma pandemia: a terceira onda da Covid-19 ou o paciente invisível. InO lado oculto de uma pandemia: a terceira onda da COVID-19 ou o paciente invisível. Conass 2020; https://www.conass.org.br/wp-content/uploads/2020/12/Terceira-Onda.pdf. Acessed 5 May 2021.

[38] Marques N.P., Silveira D.M., Marques N.C., Martelli D.R., Oliveira E.A., Martelli-Júnior H. (2021). Cancer diagnosis in Brazil in the COVID-19 era. Semin Oncol.

[39] Verba menor para saúde em 2021 coloca SUS em risco, dizem especialistas. https://valor.globo.com/brasil/noticia/2020/10/16/verba-menor-para-saude-em-2021-coloca-sus-em-risco-dizem-especialistas.ghtml. Accessed March 2021.

[40] Dos Santos M.B., Pires A.L., Saporiti J.M., Kinalski M.D., Marchini L. (2021). Impact of COVID-19 pandemic on oral health procedures provided by the Brazilian public health system: COVID-19 and oral health in Brazil. Health Policy Technol.

[41] da Cunha A.R., Antunes J.L., Martins M.D., Petti S., Hugo FN. (2021). The impact of the COVID-19 pandemic on hospitalizations for oral and oropharyngeal cancer in Brazil. Community Dent Oral Epidemiol.

[42] Rocco B., Sighinolfi M.C., Sandri M. (2020). The dramatic COVID-19 outbreak in Italy is responsible of a huge drop in urological surgical activity: a multicenter observational study. BJU Int.

[43] Valdivia A.R., San Norberto E., Moreno R. (2021). Massive drop in elective and urgent aortic procedures during the peak of the COVID-19 outbreak in Spanish multicenter analysis. J Vasc Surg.

[44] Wang Y.H., Bychkov A., Chakrabarti I. (2020). Impact of the COVID-19 pandemic on cytology practice: an international survey in the Asia-Pacific region. Cancer Cytopathol.

[45] Chou A.C., Akagi R., Chiu J.C., Han S., Lie DT. (2020). The impact of coronavirus disease 2019 (COVID-19) on orthopaedic practice: perspectives from Asia-Pacific. Transient J Trauma Orthop Coronavirus.

[46] Gupta M., Pawar N.M., Velaga NR. (2021). Impact of lockdown and change in mobility patterns on road fatalities during COVID-19 pandemic. Transp Lett.

[47] Mortes na pandemia, Mortes no trânsito: por que a Covid-19 reforça a urgência da segurança viária. https://wribrasil.org.br/pt/blog/2020/05/mortes-na-pandemia-mortes-no-transito-covid-19-reforca-urgencia-da-seguranca-viaria. Accessed January 2021.

[48] Garcia VD, Pêgo-Fernandes PM. Organ transplantation and COVID-19. Sao Paulo Medical Journal. 2021;139:301-4. 10.1590/1516-3180.2021.139420052021 PMC9615585 34190892

[49] Castro M.C., Han Q.C., Carvalho L.R., Victora C.G., França GV. (2018). Implications of Zika virus and congenital Zika syndrome for the number of live births in Brazil. Proc Natl Acad Sci.

[50] Luppi F, Arpino B, Rosina A (2020 Jun). There is no evidence of a COVID-19 baby boom in Europe–but there is of a bust. British Politics and Policy at LSE.

[51] Lindberg L.D., VandeVusse A., Mueller J., Kirstein M. (2020).

[52] Been J.V., Ochoa L.B., Bertens L.C., Schoenmakers S., Steegers E.A., Reiss IK. (2020). Impact of COVID-19 mitigation measures on the incidence of preterm birth: a national quasi-experimental study. Lancet Public Health.

[53] Babu T.A., Sharmila V., Bhat BV. (2020). Curious scenario of changes in incidence of preterm births during COVID-19 pandemic. Pointers for future research?. Eur J Obstet Gynecol Reprod Biol.

[54] Decree N° 263, Ministry of Health. February 22, 2019. https://www.in.gov.br/materia/-/asset_publisher/Kujrw0TZC2Mb/content/id/68365657. Accessed January 2021.

[55] Vacinômetro COVID-19, Ministério da Saúde. https://infoms.saude.gov.br/extensions/DEMAS_C19_Vacina_v2/DEMAS_C19_Vacina_v2.html. Accessed December 2021.

[56] Estatísticas da Federação Nacional de Saúde Suplementar, https://fenasaude.org.br/estatisticas/beneficiarios.html#tab1. Accessed December 2021.

