# Supplementary material for: Brazil's health system functionality amidst of the COVID-19 pandemic: An analysis of resilience
Source: Lancet Reg Health Am. 2022 Mar 5;10:100222. doi: 10.1016/j.lana.2022.100222 (PMC8896985; doi:10.1016/j.lana.2022.100222)
Supplement: Supplementary file 1 [file mmc1.docx]

Supplementary Table 1. SIGTAP* main groups used to create the categories presents in our study

| Screening | Cytopathological | (0203) |
| --- | --- | --- |
|  | Diagnostics by Ultrassonography | (0205) |
|  | Diagnostic by Endoscopy | (0209) |
|  | Diagnostic by Radiological Intervention | (0210) |
| Irrepressible | Oncological Treatments | (0305) |
|  | Nephrological Treatments | (0304) |
| Phys. Appoint. | Physicians Appointments | (0301) |
|  | Clinical Treatments | (0303) |
| Surgeries LMC | All surgeries that don’t require hospital admission | (04)[SIA] |
| Surgeries HC | All surgeries that require hospital admission | (04)[SIH] |
| Transplants | Organ transplantation | (0505) |
| Diagnostic | Radiological Diagnostics | (0204) |
|  | Diagnostic by MRI | (0207) |
|  | Diagnostic by Tomography | (0206) |
|  | Diagnostic by rapid tests | (0214) |
| Ext. Causes | Clinical treatments due to external causes | (0308) |
| Childbirths | Childbirths | (0310) |
| Other | All other non-administrative and non-covid categories | (*) |

*http://sigtap.datasus.gov.br/tabela-unificada/app/sec/inicio.jsp
